# A Treatise on Diseases of the Chest

**Published:** 1822-03-01

**Authors:** 


*A Treatise on the Diseases of the Chest, in which they are described according to their Anatomical Characters, and their Diagnosis established on a new Principle by Means of Acoustick Instruments. With Plates.* Translated from the French of R. T. H. Laennec, M. D. with a Preface and Notes by John Forbes, M. D. Physician to the Penzance Dispensary, Secretary of the Royal Geological Society of Cornwall, &c. &c. One volume, 8vo, pp. 438, eight copper plates. London, 1821.

About half of our present readers will remember that, in the seventh number of our *quarterly* series, for January 1820, we gave an extended analysis of Laennec in the original. As we then predicted, the work has since excited much attention in this country, and is now put into a form and language that will facilitate its diffusion among all classes of medical society on this side of the channel. Dr. Forbes is entitled to much praise for the laborious task he has imposed on himself of re-modelling the arrangement of the original work, and concentrating the language, so as to occupy but one volume instead of two—a double improvement, that saves both time and expense. He has also enriched the work by the addition of a sensible preface, and many valuable notes. From the preface we shall make one short extract, illustrating the earnest and zealous manner in which Dr. Forbes enforces his precepts.

"Although not strictly within the scope of the present remarks, there is one other point in the diagnosis of internal diseases which I would beg leave to impress upon the minds of the younger part of the profession as of the greatest importance. Of this I have always been convinced; but it is at present more immediately and more forcibly suggested by the consideration of the measures of M. Laennec. What I allude to is—the examination of the external parts of the body in the case of internal disease. How often have I known plain and obvious diseases entirely mistaken and mistreated for months,—even years,—merely from the practitioner's neglecting this simple but necessary measure! In every case of disease, whether its seat be in the head, trunk, or extremities, we ought to examine the suspected part freed from covering, or at least from every species of covering that can impede the necessary examination,—always by the hand, and often by the eye; and wherever the case is at all doubtful, we must endeavour to overcome the repugnance of our patients to the measure, however great this may be, and however natural and proper we may feel it to be, in certain individuals. In this endeavour, if properly conducted, I may venture to say that we shall rarely fail. From the neglect of this precaution I have known peritonitis and enteritis mistaken for simple colic;—disease of the heart for disease of the stomach; and derangements depending on curvature of the spine treated for years as a mere nervous affection, and, in other cases, as organic disease of the heart, lungs, or diaphragm!

——————— vestis adempta est;
Qua posita, nudo patuit cum corpore crimen." P. xvi.

We have this very day (18th December) seen a striking example of these kinds of mistakes, where a physician, of no mean name and note, treated a gentleman a long time, as having gall-stones, though, on examination of the abdomen, it was abundantly evident, (and we had the assistance of Mr. Shaw's anatomical skill in the examination) that the complaint was a mechanical obstruction in the *caput coli*. The whole of Dr. Forbes's preface we recommend to the careful perusal of every young practitioner. The circumstance of our having reviewed the original work will necessarily limit our present notice of the translation. Yet, as the circulation of our Journal has more than doubled since our analysis of Læennec's work appeared, and as we then passed very rapidly over several important portions of the volume, we shall probably be excused for occupying a few pages of the present series with a slight sketch of some of the many highly interesting subjects embraced in the work under consideration.

The chapters which treat of the pathology of the *pleura*, are exceedingly interesting, and were those which we analysed with least minuteness in our former article; we shall therefore select them for the principal theme of the present paper.

*Acute Pleuritis*. By this our author means inflammation of the pleura itself, and does not, as is sometimes done, confound it with pulmonic inflammation. There is no doubt that pleurisy and peripneumony often co-exist—that the stitch in the side is sometimes altogether wanting in pleuritis—and that the said stitch is very acute, occasionally, where violent peripneumony is combined with slight pleurisy. Still, these two inflammations may, and do exist, singly. We more frequently find, on dissection, peripneumony without pleurisy, than pleurisy without peripneumony—a fact which may be accounted for, by simple pleurisy being always cured.

" Pleurisy is either chronic or acute. The anatomical character of acute pleurisy, like that of the inflammation of all serous membranes, is redness of the part affected. This redness is in some sort punctuated, and looks as if one had traced with a pencil upon the pleura an infinity of small bloody spots of very irregular figure, and very close to one another. These red points occupy the whole thickness of the membrane, and leave small intermediate portions retaining the natural white colour. This punctuated appearance is unquestionably a character of the inflammation, and not at all attributable, as some have supposed, to the partial disappearance of the redness after death. Besides this particular redness,—and even in those instances, where it is very inconsiderable,—we always find the superficial blood-vessels of the pleura redder, more distinct, and more distended than in the natural state." 147.

M. Laennec never could clearly make out a distinct thickening of the pleura in these cases—such supposed thickening, he thinks, has been either an extensive congeries of miliary tubercles on the outer or inner surface of the pleura—or a cartilaginous incrustation on the parts covered by it—or, lastly, false membranes, more or less dense, adherent to its internal surface.

" Inflammation of the pleura is always accompanied by an extravasation on its internal surface, and which may be considered as the species of suppuration proper to serous membranes. This extravasation appears to commence with the inflammation itself. It consists of two very different matters. The one, of a firmer, semi-concrete consistence, is usually termed *false membrane*, or coagulable lymph; the other, very thin and watery, is called, *serosity*, or *sero-purulent effusion*. Both of these exhibit great variation of character." 147.

The false membranes consist of a yellowish-white, opake, or slightly semi-transparent matter, varying from the consistence of thick pus, to that of boiled white of egg, or of the buffy coat of the blood. This substance forms a complete incrustation (where the inflammation is general) over the pleura costalis and pleura pulmonalis. These two sheets of pleura are sometimes united by bands of the same membrane, extending from the one to the other, through the serous fluid effused into the cavity. These membranous exudations vary in thickness, from half a line to two lines—sometimes exhibiting a kind of reticulated structure—at others, appearing studded or granulated with small irregular tubercles. These membranes are sometimes detached and found floating in the serosity.

The serous effusion is commonly of a lemon, or light yellow colour, transparent, or slightly flocculent, resembling unstrained whey—an effusion common to all the serous membranes in the body. In some cases, this effusion is of a very deep tawny colour, ruddy and evidently mixed with blood—sometimes, indeed, quite bloody. The portions of pleura situated beneath false membranes, when this is the case, are much redder than in the most acute recent inflammation, owing, our author thinks, to a secondary inflammation supervening at a later period, than the formation of the false membranes. The effused fluid is generally without taste or smell in acute pleurisy. The relative proportions between the effused serum and albuminous concretions, is not at all fixed. Generally speaking, the more violent the inflammation, the more extensive and thick is the membranous exudation. In weak, leuco-phlegmatic subjects, on the contrary, we find a great quantity of limpid serum, with a small portion of thin membrane often floating in it. In such cases, the pleurisy seems to pass insensibly into hydro-thorax, as we shall see more particularly hereafter. It is an exceedingly rare case, M. Laennec observes, to find the contiguous surfaces of the pleura united, without previous effusion of fluid, since the absorption of the fluid is the first step in the sanative process. We may therefore conclude, on finding those old adhesions of the lungs to the sides, that there existed, in the acute stage of the disease, an effused fluid—for when they happen to die in that stage, we almost invariably find an effusion, as every practitioner knows. The following passage will convey a clear idea of the sanative process in pleurisy, and the organization of the extravasated substances.

"It is the character of the false membranes produced in pleurisy to be changed into cellular substance, or rather into a true serous tissue like that of the pleura; and this is the natural progress of the process when left quite undisturbed. This change is produced in the following manner: the serous effusion which accompanied the membranous exudation is absorbed, the compressed lung expands, and the false membranes investing it and the costal pleura become united into one substance. By and by, this substance becomes divided into layers pretty thick and opaque, which are separated by a very small portion of serosity. About this time blood-vessels begin to make their appearance in it, the first rudiments of which have the aspect of irregular lines of blood, much larger than the vessels which are to take their place. The blood seems as if it had been forced into the substance of the false membrane by a strong injection; and we find the corresponding portions of the pleura redder than elsewhere, and as it were spotted with blood. After a time, the pseudo-membranous layers become thinner and less opaque; the lines of blood assume a cylindrical shape, and ramify in the manner of blood-vessels, but still preserving their augmented diameter. On minutely examining these at this stage, we find their external coat consisting of blood scarcely yet concrete, and very red; within this there is a sort of mould, or rounded substance, whitish and fibrous, and formed evidently of concreted fibrine, perforated in its centre, already permeable to the blood, and evidently containing it. Eventually, the layers of the false membrane become quite transparent, and nearly as thin as those of the ordinary cellular tissue, and the blood-vessels resemble in every respect those which ramify on the inner surface of the pleura. It wants, however, the firmness of the natural cellular substance, being easily torn in our attempts to examine it, and its vessels still retain the large diameter indicative of their recent formation: and it requires some considerable time for them to attain the perfect character of the original tissues of the body." 153.

These organizations having taken place, the health remains unaffected, and the respiration, except in some particular cases, does not suffer from their presence.

In simple pleurisy, we find no sign whatever of inflammation of the pulmonary parenchyma, even in the vicinity of the most inflamed portions of pleura. We find the substance of the lungs, in such cases, more dense and less crepitous, owing to the compression of the effused fluids. If the extravasation has been very great, the lung becomes flattened, ceases to contain air, and consequently to crepitate; its vessels are compressed and contain little blood, and the bronchia, with the exception of the larger trunks, are evidently rendered smaller.*

Sometimes, however, we observe certain portions of the lungs possessing a redness like that of muscle; and a compact homogeneous texture, in which no trace of air-cell can be detected. This has been termed carnification or hepatization of the lungs. Our author considers it a product of inflammation.

Here M. Laennec notices a symptom of pleurisy accompanied by effusion—to wit, *enlargement* of the side of the chest, a dilatation noticed by all writers on empyema, since the days of Hippocrates, but which our author has ascertained to take place from effusion in recent pleurisy. He has often found it very distinct after two days illness. It is, of course, much more evident in lean, than in fat persons. On measuring the affected side with a piece of ribband, we find it enlarged, but not so much so, as to appear to the eye. In proportion as the effusion becomes absorbed, the dilatation of the chest insensibly disappears; and then, as we shall presently see, the affected side sometimes becomes narrower than before the disease.

---

* We again and again exhort our junior brethren to study these and all the other minutiæ of pathological anatomy with the utmost care, were it for no other purpose than that of being able to discriminate between morbid and healthy structure, when opening bodies. Few days pass that we have not opportunities of seeing false conclusions drawn in post mortem examinations from non-acquaintance with minute morbid anatomy.—Rev.

*Chronic Pleurisy.* This does not differ materially, in its anatomical character, from the acute form. In the chronic disease, the pleura is commonly of a deeper red—the serous effusion more abundant, and almost always less limpid, being mixed with a great quantity of albuminous flocculi, sometimes rendering the liquid quite puriform by their abundance and minuteness. In such cases, these puriform fragments accumulate in the most depending parts of the thoracic cavity, and, by their consistence, form a link between the sero-purulent effusion and the false membranes. The extravasated fluids in chronic pleurisy, are rarely so free from smell as in the acute—sometimes they have a heavy odour, more disagreeable than that of healthy pus. This disease has rarely any natural tendency towards resolution. "In cases of extravasations which have lasted several months, we find no mark of any step towards the conversion of the false membranes into cellular substance." A cure, however, is sometimes effected in a manner that shall presently be shewn.

"The effusion produced by chronic pleurisy tends, most commonly, to become daily more considerable. The affected side becomes manifestly larger. The intercostal spaces grow broader, and rise to a level with the ribs, and sometimes even higher. The lung of the affected side, compressed towards the mediastinum and spine, and retained in this position by the pseudo-membranous exudation which covers it completely, is sometimes reduced to a thickness of little more than half an inch, even in its middle, and, without a careful examination, might be considered as totally destroyed. In this state the pulmonary tissue is soft, pliant, and dense, like a piece of leather, without any crepitation, more pale than natural, occasionally greyish, and entirely without blood. Indeed the blood-vessels are often seen flattened and empty. The cellular texture is nevertheless still very distinct; and sometimes, though rarely, some points are found in the state of *carnification* above described. This case constitutes the *Empyema* of authors, at least of modern authors; for I apprehend no one now considers empyema as the product of a vomica which has burst into the cavity of the pleura. A softened tubercle may, indeed, discharge its contents in this manner, and may thus become the cause of a considerable effusion by exciting a chronic pleurisy, but in such a case the tuberculous matter must only be considered in the light of an extraneous body determining inflammation, and consequent effusion, by its mechanical or chemical qualities. It is also to this species of pleurisy that we must refer those histories of *lungs entirely destroyed by suppuration* which we find recorded in the older writers." 158.

Such is chronic pleurisy. It exhibits, in none of its stages, the intense fever, re-action, and acute pain, that characterize an active disease. It commonly attacks subjects of a worn-out constitution, more especially such as have suffered from a tubercular affection of the lungs or other organ. This complication with other diseases, and want of prominence in its symptoms, general and local, cause it too often to be overlooked, or misunderstood. There is another variety of chronic pleurisy, namely, where the acute disease becomes chronic from any cause which prevents the absorption of the effused fluids, and the conversion of the false membranes into cellular substance. Hydrothorax is a result of this state, and will be considered farther on.

Before we proceed to the section on "contraction of the chest, consequent on certain pleurisies," we shall take some notice of the *diagnosis* of pleurisy, which Dr. Forbes has *translated* to another part of the volume.

Well-marked acute pleurisy is easily recognized in general. The stitch in the side, dyspnœa, fever, dry cough, or cough accompanied only by glairy and almost colourless sputa, are often sufficient for the diagnosis.

"But it is not uncommon to meet with pleurisies, even acute, in which many of these symptoms are wanting; whilst many chronic pleurisies, are often so indistinctly characterised, and accompanied by so many functional anomalies, that it is frequently not till after several weeks, or even months, that the true nature of the disease comes to be suspected." 333.

Percussion points out the disease with more certainty. Resonance of the chest fails, over that part where the effusion exists. This failure, it is true, may also arise from peripneumony; but the general symptoms, and particularly the expectoration, will tend to fix the distinction. The application of the stethoscope affords still more certain means of discrimination. The signs by which the cylinder effects this, are, 1st, the total absence or great diminution of the respiratory murmur, heard in a sound state—2dly, the appearance, disappearance, and return of the sound, to which our author has given the term *Hœgophonism*.

"When, as it is often the case, the pleuritic effusion is very copious from its very commencement, the sound of respiration is then totally absent through the whole of the side affected, except in the space of three finger's breadth along the vertebral column. This complete disappearance of respiration after the existence of disease for a few hours, is quite pathognomonic of pleurisy with copious effusion." 334.

In peripneumony, the cessation of the respiratory murmur is gradual, and is perceived to be unequal in different parts of the chest, being often not lost in the upper part, till after some days or weeks; in pleurisy, on the contrary, the loss of the respiratory murmur is sudden, equable, uniform, and so complete, that no effort of inspiration can render it perceptible.

These copious and sudden effusions occur chiefly in old persons, or in adults of weak and cachectic habits. The sudden cessation of the respiratory noise through the cylinder, must therefore be considered a bad prognostic, as we may be assured that, the conversion of the false membranes into cellular substance, and the absorption of the fluid will either not at all take place, or imperfectly, and, consequently, that the disease will soon pass into the chronic state.

"When the effusion begins to diminish, by absorption, this is first observable by the augmented intensity of the respiratory murmur along the side of the spine, where it had never quite disappeared. Shortly after, it is perceptible in the anterior superior part of the chest, and top of the shoulder, and in a few days it returns in the other parts of the chest. Wherever there are adhesions between the lungs and pleura, of any considerable extent, the respiration continues audible over them in a greater or less degree throughout the whole period of effusion; and the commencement of the absorption is perceived by the augmented intensity of sound in these places." P. 335.

*Contraction of the Chest consequent on certain Pleurisies.* There are some cases of pleurisy wherein the affected side never becomes sonorous, in the trial of percussion, although the disease has been cured, and the effused fluid absorbed. Cases of this kind, though not very rare, have not attracted much attention. The subjects of this morbid alteration are easily distinguishable by their external shape and gait—they seem always to lean towards the affected side, which is manifestly narrower than the other, there being, frequently, more than an inch of difference, when they are measured by a cord. The length of the chest is also diminished—the ribs are closer together—the shoulder is lower,—and the muscles, especially the pectoral, are only half the size of those on the opposite side.

"The difference of the two sides is so remarkable, that, at first sight, we should think it much greater than it is found to be by admeasurement. The spinal column generally remains straight; sometimes, however, it at length yields through the effect of the habitual leaning towards the diseased side. This habit gives to the individual the appearance of being somewhat lame." P. 160.

This morbid contraction of one of the thoracic cavities arises from a somewhat irregular termination of chronic pleurisy, or acute pleurisy, become chronic. In these cases the seropurulent effusion having continued a long time, the false membranes investing the pleura and lungs acquire a particular hardness, and an incipient organization which render them incapable of being converted into cellular substance. When the effusion is absorbed, the lung long compressed by it, and further bound down by a strong false membrane, completely investing it, cannot dilate itself promptly enough to keep pace with the progress of the absorption; the ribs consequently contract, and the cavity of the chest is thus diminished. When the fluids are entirely absorbed, the costal and pulmonary exudations come into contact and finally unite, so as to form only one substance, the consistence of which becomes daily firmer, till, in a few months, it acquires the character of a fibrous, or fibro-cartilaginous membrane.

These membranes have been commonly described, our author thinks, under the name of *thickenings* of the pleura—a mistake very liable to be made by those who trust to the mere appearance of these, without further examination. But on dissection we can always detach them from the pleura, which is found of its natural thickness.

This contraction of the chest coinciding with the absorption of the serous effusion, is frequently not perceptible till after several months of disease, or tedious convalescence. At length, however, the patient regains perfect and often permanent health. Two plates are given, representing back and front views of patients with this curious contraction. M. Laennec is deserving of great praise for his minute and curious pathological investigation of this interesting subject.

*Hydrothorax*. M. Laennec thinks that *idiopathic* hydrothorax is an extremely rare disease.* Many other disorders are often ranged under the head of hydrothorax, as diseases of the heart and great vessels, irregular consumptions, and even scirrhus of the stomach and liver. What has chiefly led to the idea of hydrothorax being a frequent disease, is the common mistake of taking a *sero-purulent effusion* for it—a part of these effusions being generally transparent.

---

" * The great rarity of the true hydrothorax ought to make us cautious how we give this name to so many affections as we are accustomed to do; and the undoubted fact of a serous effusion being an almost uniform attendant on the inflammation of serous membranes, ought to make us slow to trust to mere diuretics and other similar remedies in cases wherein we have strong reason for suspecting dropsical effusion, especially in the chest. The now very generally allowed connexion between dropsy and inflammation, mentioned by our author in many parts of this treatise, is still much better understood in England than France. It is therefore hardly necessary to refer the English reader to the works of Blackall and Parry, and especially Crampton, for the practical and pathological illustration of this important doctrine." P. 429.

"Idiopathic hydrothorax commonly exists only on one side. Its anatomical characters are simply an accumulation of serum in the cavity of the pleura; this membrane being quite healthy in other respects; and the lung being compressed towards the mediastinum, flaccid, and destitute of air, as in cases of pleuritic effusion. When the effusion is very great, the affected side is evidently larger than the other. This disease may exist in a very great degree without any other symptom of dropsy in any other part of the body." 193.

The chief, almost the only symptom of this disease, is impeded respiration—its progress, and the state of the general symptoms, can alone distinguish it from chronic pleurisy.

*Symptomatic Hydrothorax** is as frequent as the idiopathic is rare. The symptomatic effusion may accompany almost every disease, acute or chronic, local or general, usually announcing their fatal termination. It is not, perhaps, more frequent in cases of ascites and anasarca than in other diseases. It is commonly met with in persons dead of acute fevers, diseases of the heart, or tubercles or cancer of different organs.

"Its symptoms, which are in every respect like those of the idiopathic disease, do not, in general, make their appearance but a few days, or even hours, before death. When the effusion takes place on both sides of the chest, it produces a very painful suffocation. Sometimes, however, we find a considerable effusion in both sides, in cases where there had been no very notable dyspnœa before death. Might not the effusion in such cases take place in the very moment of dissolution,—or even after death? We know that the functions of the capillary system do not cease immediately after death. The quantity of serum effused varies from a few ounces to one or two pints. It is commonly colourless or yellowish, sometimes tawny, reddish, or even bloody." 195.

There are organic affections of the pleura which give rise to hydrothorax. These are cancerous or tuberculous degenerations. The first are commonly of the variety called medullary or soft cancer, of varying size, but rarely bigger than an almond. They adhere strongly to the pleura, and have the usual characters of medullary fungus. The tuber-

---

"* In this and many other parts of the treatise, our author notices the tuberculous affections of serous membranes lately so ably illustrated by Dr. Baron. It must be very satisfactory to that gentleman to have his statements corroborated by so great an authority; more especially as they were evidently unacquainted with each other's inquiries. Dr. Baron's work is a most valuable addition to our pathological knowledge; although the author appears occasionally to have extended his peculiar views to some morbid appearances which might perhaps be explained on the principle of ordinary inflammation." P. 429.

cles that form on the surface of the pleura are generally very numerous, varying in size from a millet to a hemp seed. They are often united by means of a soft semi-transparent false membrane. At an early period we can sometimes scrape off this false membrane, and with it the greater number of the tubercles, which are evidently rather developed in it than in the pleura itself. At a later period the false membrane disappears, or unites with the pleura—which then seems thicker, in which case the tubercles appear embedded in its substance.

"We find also, occasionally, on the pleura, another variety of granulations which resemble some other cutaneous eruptions. This consists of small, white, opaque, flattened grains, placed very close together, and of a firm texture like that of fibrous membranes. This variety is also accompanied by thickening of the pleura. It appears to me to be the result of the imperfect organization of a false membrane, such as I have already described (page 147.) These two varieties of morbid growths are very rare on the pleura, but very frequent on the peritoneum. Bichat is the first who noticed them, though he does not seem to have known the real nature of them: they are always accompanied by hydrothorax." 179.

Our readers will recognize, in these passages, what our countryman, Dr. Baron, has since more fully developed under the title of "tuberculated accretions of the serous membranes."

*Pneumo-Thorax.** Occasionally we find aeriform fluids in the cavities of the pleura, which are sometimes without smell, but more commonly fetid, resembling sulphuretted hydrogen gas. These fluids are occasionally in such quantity as very forcibly to compress the lung, and distend the thoracic parietes. In this case the ribs are found more or less separated, and the diaphragm projecting into the cavity of the abdomen, forcing the viscera before it.

Although not exceedingly rare, this affection has been but little observed by medical men. In general we know it only by the casual observations of anatomists and surgeons, who have occasionally noticed the escape of air in opening the chest after death, or in performing the operation of em-

---

"* Dr. Duncan, Jun. informs me that he has often met with this affection in cases of empyema. Where he suspects it before opening the thorax, he examines the diaphragm from the abdomen.—'In one case lately,' he says, 'as I predicted, we found the diaphragm on one side convex upwards, and on the other convex downwards: on puncturing an intercostal space on *this* side, the air rushed *out* and the diaphragm rose into the chest." 429.

pyema. M. Itard, in an inaugural dissertation on pneumothorax, has detailed five cases of the disease—three of which are original, one extracted from Selle, and one from M. Bayle. In all these the aerial effusion co-existed with phthisis and chronic pleurisy. In all of them the lung of the affected side was compressed into a small compass at the root—the fluid was more or less fetid—the cavity of the pleura was lined by a false puriform membrane, and contained a few spoonfuls of pus.

We must close our very imperfect notice of Dr. Forbes's translation, by a short abstract of his 4th chapter, part the first, on hæmoptysis, or pulmonary apoplexy, as it has been not inaptly termed by M. Laennec, and some other continental writers.

Both antients and moderns have attributed hæmoptysis to a rupture of some of the pulmonary vessels; but this pretended cause of the hæmorrhage is now proved to be altogether false. Two varieties of hæmoptysis may, however, arise from this rupture of vessels, namely, *first*, where an aneurism bursts into the bronchia, or trachea; and *secondly*, where there is a rupture of a blood-vessel into a tuberculous excavation—an event extremely uncommon. These two species are almost immediately fatal, and can by no means explain the phenomena of a disease so common, and often so slight as hæmoptysis.

"Accordingly, hæmoptysis is now very generally considered as depending on some functional derangement of the bronchial membrane, which causes it to exhale blood in place of its ordinary mucous secretion. And this opinion is unquestionably correct as far as regards the slighter varieties of the disease, such, for instance, as occur in pulmonary catarrh, peripneumony, and in the earlier stages of phthisis." P. 61.

Those cases, however, of violent and extreme hæmorrhage which often resist all treatment, arise from a very different and more dangerous cause than mere exhalation from the bronchial membrane.

"In these, some part of the pulmonary substance has undergone great changes, being indurated to a degree equal to the completest hepatization. The induration, however, is very different from the inflammatory affection of the lungs distinguished by this term. It is always partial, and never occupies a considerable portion of the lungs; its more ordinary extent being from one to four cubic inches. It is always very exactly circumscribed, the induration being as considerable at the very point of termination as in the centre. The pulmonary tissue around is quite sound and crepitous, and has no appearance whatever of that progressive induration found in the peripneumonic affection. The substance of the lung is, indeed, often very pale around the hæmoptysical induration; sometimes, however, it is rose-coloured, or even red, as if tinged with fresh blood; but, even in this case, the circumscription of the indurated part is equally distinct.

"The indurated portion is of a very dark red, exactly like that of a clot of venous blood. When cut into, the surface of the incisions is granulated as in a hepatised lung; but in their other characters, these two kinds of pulmonic induration are entirely different. In the second degree of hepatisation, along with the red colour of the inflamed pulmonary tissue, we can perceive distinctly the dark pulmonary spots, the blood-vessels, and the fine cellular intersections, all of which together give to this morbid state the aspect of certain kinds of granite, as has been already observed. The same thing is observable in the third stage of peripneumony, and even when the infiltration of pus has converted the lungs into a yellowish mass. In the induration of hæmoptysis, on the contrary, the diseased part appears quite homogeneous, being altogether black, or of a very deep brown, and disclosing nothing of the natural texture of the part, except the bronchial tubes and the larger blood-vessels. The latter have even lost their natural colour, and are stained with blood." P. 62.

In scraping the incised surfaces of these parts, we detach a small portion of very dark half congealed blood, but in a much less proportion than we can press out the bloody serum from a hepatized lung. Sometimes the centre of these indurated masses is soft, and filled with a clot of pure blood.

This morbid affection appears to our author to be evidently produced by an effusion of blood into the parenchyma of the lungs—in other words, into the air cells. From its exact resemblance to the effusion that takes place in the brain in apoplexy, M. Laennec has given it the term pulmonary apoplexy. The lungs and brain, however, are not the only organs in which a similar effusion may occur. It is seen to take place instantaneously in the subcutaneous cellular tissue between the intestinal and ocular tunics, among the muscular fibres of the heart, and under the cellular coverings of the pancreas and kidneys. "In a case of fatal apoplexy, says M. Laennec, I have found large effusions of blood in the cellular membrane of every limb, of the trunk, and in that surrounding most of the abdominal viscera."

"The hæmorrhagic induration of the lungs is as easily distinguishable from the congestions that take place after death, as from the alterations produced by peripneumony. The sanguineous congestions of the dead body consist of an accumulation of blood intermixed with serum, often spumous, which flows plentifully on an incision of the part, and tinges the lungs of a livid or vinous colour. Being the mere consequence of gravitation, the engorgement is found most considerable in the most depending parts of the lungs, and gradually lessens towards the superior parts. Where most engorged, the part still retains some crepitation, and the incised surfaces are never granulated, even when the congestion is so great as to destroy the spongy character of the lung. By washing, we can, in every case remove all the blood, and restore the lung to that sort of flaccidity which it possesses when compressed by a pleuritic effusion. The engorgement of hæmoptysis, on the contrary, is accurately circumscribed, very dense, dark-red or brown, granulated, and almost dry when incised, and grows pale by washing, but without losing any part of its consistence." P. 64.

Whatever may be the severity of the case, resolution seems to take place with considerable facility, since we find a great many instances of cure after very severe hæmoptysis. The above is the condition of parts in all these severe cases; but when the symptoms are moderate, and the hæmorrhage slight, the only morbid alteration of structure is a reddening and thickening of the bronchial membrane, which, in these cases, seems to permit the transudation of the blood.

Therapeutical matters enter not into the composition of this valuable work; but we may just remark, en passant, that we have found practitioners in this country too timorous in respect to the exhibition of superacetate of lead and opium in hæmoptysis. We have happened to be much in the way of this disease, and we have found such decided benefit from the combination abovementioned, and that in large doses, that we beg to impress the circumstance on the memory of our younger brethren. Without having so accurate an idea of the real pathology of the disease as Laennec has since imparted, we had long been convinced that the blood came from the capillary vessels, and consequently that those remedies which are known to have an influence on the capillary system, were indicated. We have often seen men bled day after day, and take digitalis, infusion of roses, and sulphuric acid, in hæmoptysis, without materially lessening the flow from the lungs; while, on the other hand, a moderate bleeding, followed by superacetate of lead and opium, has restrained effectually some of the most alarming discharges of blood which we ever witnessed.

But we must now reluctantly take leave of Dr. Laennec and his translator. To the former we have often paid the tribute of our respect for his talents, and unwearied exertions in the science of pathology. In Dr. Forbes the public has a physician of native genius and acquired knowledge—the profession, a member of zeal, honour, and integrity.

